# Acute aortic dissection: be aware of misdiagnosis

**DOI:** 10.1186/1756-0500-2-25

**Published:** 2009-02-20

**Authors:** Irene Asouhidou, Theodora Asteri

**Affiliations:** 1 Department of Cardiac Anesthesia, "G. Papanikolaou" General Hospital, Exohi, Thessaloniki, Greece

## Abstract

**Background:**

Acute aortic dissection (AAD) is a life-threatening condition requiring immediate assessment and therapy. A patient suffering from AAD often presents with an insignificant or irrelevant medical history, giving rise to possible misdiagnosis. The aim of this retrospective study is to address the problem of misdiagnosing AD and the different imaging studies used.

**Methods:**

From January 2000 to December 2004, 49 patients (41 men and 8 women, aged from 18–75 years old) presented to the Emergency Department of our hospital for different reasons and finally diagnosed with AAD. Fifteen of those patients suffered from arterial hypertension, one from giant cell arteritis and another patient from Marfan's syndrome. The diagnosis of AAD was made by chest X-ray, contrast enhanced computed tomography (CT), transthoracic echocardiography (TTE) and coronary angiography.

**Results:**

Initial misdiagnosis occurred in fifteen patients (31%) later found to be suffering from AAD. The misdiagnosis was myocardial infarction in 12 patients and cerebral infarction in another three patients.

**Conclusion:**

Aortic dissection may present with a variety of clinical manifestations, like syncope, chest pain, anuria, pulse deficits, abdominal pain, back pain, or acute congestive heart failure. Nearly a third of the patients found to be suffering from AD, were initially otherwise diagnosed. Key in the management of acute aortic dissection is to maintain a high level of suspicion for this diagnosis.

## Background

Acute aortic dissection (AAD) is a potentially fatal condition that requires rapid assessment and treatment. However, despite major advances in imaging modalities and noninvasive studies, correct diagnosis is not always the rule, with misdiagnosis occurring in less than half the cases [1,2].

Classically, acute aortic dissection usually presents with an abrupt onset of severe pain in the chest, back, or abdomen. Patients often describe their pain as tearing or ripping. The severity of pain may precipitate vagal reflexes, including hypotension and bradycardia. If the aortic valve or coronary arteries are involved, congestive heart failure may acutely develop. The patient may present with paralysis of upper and/or lower extremities [3], anuria, dyspnea or pulmonary edema. Additionaly, most patients suffering from AAD often reveal insignificant medical history. The absence of suspicion rising from the patients medical history together with the heterogeneity of the clinical features of AD may lead to misdiagnosis.

Even though certain imaging modalities have been proven helpful in diagnosing AAD, for instance helical computed tomography (CT), magnetic resonance imaging (MRI), coronary angiography and transesophageal echocardiography (TEE), they are not always at hand in the Emergency Room. Moreover some of them are time-consuming or not applicable for hemodynamic unstable patients [2,4]. Since the progress of complications is time-dependent in cases of AAD, choosing the appropriate diagnostic test may prove crucial for the survival of these patients. The aim of this retrospective study is to address the problem of misdiagnosis in AAD, regarding the clinical features, and evaluate the accuracy of the diagnostic studies used.

## Method

This study was approved by local Ethics Committee from our hospital. Data from 49 cases of AAD were retrospectively reviewed. From January 2000 to December 2004, 41 men and 8 women referred and/or admitted to our hospital were eventually diagnosed with AAD. The patient's age ranged from 18 to 75 years, with a mean of 54.8 ± 9.2 years. The demographics data of patients who were diagnosed with aortic dissection are showed in Table 1.

**Table 1:** Demographics and history of patients (N = 49)

	n = 49	Type I n = 29	Type II n = 14	Type III n = 6
**Age **(years)	54.8 ± -9.2			

**Age < 19**		1	3	4
**Age: 20–69**		22	7	
**Age > 70**		6	4	2

**Sex**				
**Male**	41 (84%)	17	19	5
**female**	9 (16%)	2	6	1

**Ethnicity**				
**White**	49 (100%)			

**Arterial Hypertension**	15 (31%)	6	8	1

**Marfan syndrome**	1 (2%)	1		

**Horton's arteritis**	1 (2%)		1	

**Prior cardiac surgery**	0 (0%)			

**Hyperuricemia**	2 (4%)	1	1	

**Smoking**	8 (16%)	5	3	

**Hyperlipidemia**	7 (14%)	3	4	

**Diabetes mellitus**	3 (6%)	2	1	

**COPD**	3 (6%)	1	2	

COPD: Chronic obstructive pulmonary disease

From their medical history, fifteen patients were found suffering from arterial hypertension, 1 patient from giant cell arteritis (Horton's arteritis) and 1 patient suffering from Marfan's syndrome. Two patients were transported intubated, so it was impossible to obtain any information from their medical history. Thirty patients were submitted with no significant history (Table 1). Ten patients on arrival to the ER were hemodynamically unstable.

Upon admission to the ER, a detailed medical history was taken and complete physical examination performed, whenever this was possible. Electrocardiogram (ECG) and chest X-ray and blood test were performed in all patients. Depending on the clinical picture and the hemodynamic status of the patient, transthoracic echocardiography (TTE), and/or straightforward contrast enhanced computed tomography (CT) and coronary angiography were consequently performed. Transesophageal echocardiography was not available in the ER.

## Results

According to the De Bakey classification, aortic dissection Type I was identified in 29 patients, Type II in 14 patients and Type III in 6 patients [5].

Thirty five patients (71,4%) were presented with chest pain as the most common symptom, while 18 patients (36,7%) admitted to the ER with chest pain being the sole symptom (Table 2). In 17 patients chest pain was complicated by back pain (n = 4), syncope (n = 4), congestive heart failure (n = 3), or neurologic deficit (n = 6) (paralysis of lower extremities or upper extremities). Though, anterior chest pain was typical in patients with Type I dissection.

**Table 2:** Presenting symptoms

**Symptoms**	Type I (n = 29)	Type II (n = 14)	Type III (n = 6)	Presented	
				No	%

**Chest pain (total)**				**35**	**71,4%**

only chest pain	12	6		18	36,7%

Chest pain with back pain	1	1	2	4	8,1%

Chest pain with syncope	4			4	8,1%

Chest pain with neurologic deficit	2	4		6	12,2%

Chest pain with CHF	3			3	6,1%

CHF	4	1		5	10,2%

syncope	2	1		3	6,1%

Syncope with pulselessnes of the lower extremities		1		1	2%

Intubated			2	2	4,1%

paralysis of lower extremities	1		1	2	4,1%

Hemiparesis			1	1	2%

**Total**	**29**	**14**	**6**	**49**	**100%**

Hemodynamic instability	7	1	2	10	20,4%

CHF:congestive heart failure

According to diagnostic tests, chest X-ray was routinely performed in all patients and revealed a widened mediastinum in 20 patients (41%). Computer tomography was performed as the initial study in 76% (n = 37) and successfully established the dissection in all patients that it was performed. However, in 13 patients (26,5%) CT was not able to evaluate the participation of the aortic valve in the aortic dissection and define the type of dissection. Transthoracic echocardiography was performed in 49% (n = 24) of patients. Findings from TTE studies were positive in 50% of patients and false negative in 50%. Coronary angiography was used as the initial imaging study in 3 patients and it was used as second-line imaging study in 39% (n = 19) of patients. In Table 3 all imaging studies and their accuracy in establishing the diagnosis of AD are depicted in detail.

**Table 3:** Findings on image studies

**Study**	Type I (n = 29)	Type II (n = 14)	Type III (n = 6)	Presented,	
				No	%

Electrocardiography	29	14	6	49	100
abnormal	1				

Chest x-ray	29	14	6	49	100
Wide mediastinum	14	5	1	20	41

TTE	17	6	1	24	49
true positive	11	1		12	50
false negative	6	5	1	12	50

CT	25	8	4	37	76
true positive	25	8	4	37	100

Coronary angiography				22	11
true positive				21	95,45
false negative				1	

TTE: Transthoracic echocardiography, CT: Computed tomography

Cardiac troponin was tested in 12 patients suspicious for myocardial infarction and turned positive in only one of them.

A total of 15 patients (31%) later found to be suffering from an AAD were initially otherwise diagnosed.

Twelve patients, with normal TTE exam, were for a variety of reasons initially diagnosed with myocardial infarction. One patient had a positive cardiac troponin test and one patient had elevation ST segment at V_4–6_. Chest X-ray revealed wide mediastinum in 7 patients (58%). Thrombolysis didn't administer in none of 12 patients: emergency coronary angiography performed in 3 patients and AAD was revealed. Emergency CT and/or coronary angiography was performed to the other 9 patients during the first hour due to sudden deteriorating. None of them died before the correct diagnosis of AAD is established and fortunately they were not affected from the delay in diagnosis.

Three patients later found to be suffering an AAD were initially diagnosed with cerebral infarction. These patients presented to the ED without any chest or back pain. Two of them reported acute onset of lower limb weakness and numbness. On physical examination, strength was 5/5 in both upper extremities and 0/5 in both lower extremities while deep tendon reflexes of lower extremities were absent. The third patient presented with left hemiparesis. All 3 patients had only slightly elevated blood pressure. Blood tests, X-rays and TTE studies were normal in all 3 patients, and they were therefore treated for cerebrovascular ischemic disease. In the first two cases the correct diagnosis of AAD was established a few hours later with CT, however in the third case the diagnosis was delayed until two days later. Due to the fact that his condition was steadily deteriorating an emergency CT was performed which showed the present of AAD

## Discussion

Aortic dissection has a myriad of clinical presentations and is for certain a diagnostic challenge. According to the relevant literature, in cases of AAD the most common symptom by far is chest pain, which is usually sharp and sometimes reported as tearing or ripping, while often radiating to the back or the abdomen [4,6]. In our case series, the acute onset of severe chest pain was the most common initial complain and in 36% of patients it was the only symptom. Chest pain was usually accompanied by back pain, paralysis of lower or upper extremities, symptoms of congestive heart failure or syncope. Less common manifestations included symptoms of congestive heart failure, syncope, lower extremity ischemia and anuria without chest pain.

In our study the correct diagnosis of AAD was straightforward in 69% of patients, making 15 patients who were later found to have suffered an AD (31%) initially misdiagnosed. Our findings are in agreement with data from large series where up to 30% of patients with AAD, were initially given a different diagnosis [7]. According to Spittell et al in 17 patients (28%) the diagnosis of AAD was not made until post-mortem examination [8].

In the present study, twelve patients were initially diagnosed with acute myocardial infarction. According to large series, confusion of AAD with acute coronary syndrome may occur in up to 45% of cases [1,9,10]. Examination of cardiac troponin contributed to the correct diagnosis in only one patient of the twelve that were tested. According to Liang et al, results of CPK-MB and cardiac troponin could not discriminate between AAD and MI in a total of 33 patients who suffered from AAD, and this fact contributed to the misdiagnosis in more than half the cases [9]. Hansen et al underlined that the confusion of AAD with acute coronary syndrome not only does delay correct diagnosis but may prove lethal for the patient due to initiation of treatment with antithrombotic agents [1]. Kawano et al also reported a case of a patient who died of AAD while treated for MI [11]. Among the other clinical examinations that might be helpful in distinguishing MI from AAD, D-dimers is a valuable test. However, because it is highly elevated in both acute PE and acute AAD [12] we did not use it as first line test in the ER.

Although pain is the most common presenting symptom in AAD, painless acute aortic dissection may occur in approximately 5% of patients [13,14]. Syncope occurred in 8% of patients with no accompanying pain. Thus, AAD should be considered in the differential diagnosis of syncope, even in the absence of pain. Acute aortic dissection is associated with neurological sequella in as many as one third of patients [3,15,16]. Painless AAD presenting as hemiplegia or paresis is a rare phenomenon, occurring in 2% to 8% of patients [3,17,18]. Data from 1,805 patients with aortic dissection showed that 4.2% of patients presented with acute paraplegia or paraparesis [3]. Donovan et al reported a case of a 77-year-old patient who presented with paraplegia, with no chest or back pain and was diagnosed with pneumonia and paraplegia. In this case chest CT was performed on hospital day 4, and revealed a type A dissecting aneurysm extending from the aortic valve leaflets to the take off the renal arteries [19].

Regarding the imaging studies used in our series, most patients had multiple imaging studies performed. Chest X-ray had a specificity of 41% in our series. Widened mediastinum in a chest X-ray is a common finding in 60% to 90% of cases of suspected AD [7], while according to Earnest et al up to 20% of chest X-rays may be negative in patients with AAD [20]. Similarly, in 464 patients enrolled in the IRAD study, chest X-ray revealed no mediastinal widening or abnormality in aortic contour in 21.3% of patients [6].

Various imaging modalities such as conventional angiography, helical computed tomography (CT), magnetic resonance imaging (MRI), transthoracic echocardiography and transesophageal echocardiography (TEE) are available to evaluate patients with suspected aortic dissection. In the IRAD, the first diagnostic test used was computed tomography in 61% [6]. According to Sommer et al, CT, MRI, and TEE are equally reliable for the diagnosis of aortic dissection. CT of the thoracic aorta is currently the imaging study of choice for the evaluation of patients with suspected AAD, with its sensitivity and specificity reaching 100% [21]. In our unit the first diagnostic test was CT in 76% of cases, with sensitivity and specificity of 100%. However CT failed to define the exact type of AAD in 13 of 37 cases (35%) and further diagnostic investigation was needed. This was due to the fact that aortic valve insufficiency is difficult to depict using CT [4].

Magnetic resonance imaging (MRI) was not the first choice of imaging modality in our Unit, because despite its high sensitivity and specificity (mean value 95%) and accuracy in confirming aortic dissection for high risk patients [4,22], MRI has certain limitation. These are time delay, restricted ability to monitor patient during imaging [23] and inability to be performed in hemodynamically unstable patients [2]. For the above reasons we use CT as a first line imaging test. On the other hand MRI is favoured for the assessment of chronic dissection [2]

Coronary angiography was performed in 22 patients and was diagnostic in 21 of them (specificity 95,45%). Coronary angiography is time consuming and so, it can not be performed to hemodynamically unstable patients. More importantly the diagnostic accuracy of this examination is limited [24]. According to the European study, sensitivity and sensitivity of coronary angiography for the diagnosis of AAD are 88% and 94% respectively [25]. Transthoracic echocardiography managed to establish the diagnosis of AAD in only 50% of patients.

Although aortic dissection is an old disease, misdiagnosis still remains an unresolved problem as was shown in our study. The diverse manifestations of the disease together with certain limitations in imaging studies contribute to this high rate of misguided diagnosis. However due to clinical awareness and vigilance and high degree of suspicion the correct diagnosis was promptly established in all cases and all patients were submitted to the appropriate therapy. The mortality rate associated with thoracic aortic dissection is high and has recently been reported to increase by 1% to 1.4% per hour when a patient remains untreated, leading to a 68% mortality rate in the first 48 hours [2,6]. Therefore, prompt and accurate diagnosis and treatment decisions between surgical and conservative intervention are mandatory for reducing mortality among patients with clinically suspected thoracic aortic dissection [26].

Our study is certainly limited by its retrospective nature and the lack of a uniform approach to all cases as many were initially not considered AAD and were evaluated and assessed in a different manner.

## Conclusion

Since AAD is a process that may occur anywhere in the aorta, the clinical spectrum of presentation is broad and unpredictable. The initial symptoms, the course of the disease, the ECG and creatine kinase changes of AAD can be easily confused with those of acute coronary syndrome, and special attention should be given to their differentiation. More rare manifestations such as anuria, paraplegia, numbness of extremities should also raise suspicion of aortic dissection. Although the clinical features of aortic dissection have gained wider appreciation, the diagnosis still remains elusive in a substantial number of patients, necessitating clinical awareness and vigilance.

## Competing interests

The authors declare that they have no competing interests.

## Authors' contributions

AI participated in the design of the study, was the anaesthesiologist in the Emergency Room in which the patients were submitted and drafted the manuscript. ATh participated in the design of the study and was the anaesthesiologist in the Cardiothoracic Unit in which were performed the operations. All authors read and approved the final manuscript.
